# Phytochemical Compounds and Antioxidant Capacity of Tucum-Do-Cerrado (*Bactris setosa* Mart), Brazil’s Native Fruit

**DOI:** 10.3390/nu8030110

**Published:** 2016-02-23

**Authors:** Fernanda R. Rosa, Andréa F. Arruda, Egle M. A. Siqueira, Sandra F. Arruda

**Affiliations:** 1 Faculty of Medicine, Campus Umuarama, Universidade Federal de Uberlândia, 38.400-902 Uberlândia, MG, Brazil; fernandarr.nut@gmail.com; 2 Chemistry Institute, Campus Samambaia, Universidade Federal de Goiás, 74.690-900 Goiânia, GO, Brazil; arruda.crystal@gmail.com; 3 Biological Sciences Institute, Department of Cell Biology, Campus Universitário Darcy Ribeiro, Universidade de Brasília, 70.910-900 Brasília, DF, Brazil; eglemasi@gmail.com; 4 Department of Nutrition, Faculty of Health Sciences, Campus Universitário Darcy Ribeiro, Universidade de Brasília, 70.910-900 Brasília, DF, Brazil

**Keywords:** tucum-do-cerrado (*Bactris setosa* Mart), antioxidants compounds, flavanols, anthocyanins, phenolic acids, stilbenes

## Abstract

This study identified major phenolic compounds of the tucum-do-cerrado (*Bactris setosa*) peel, as well as antioxidant activity and total phytochemical compound concentration of different extracts of the peel and pulp of this fruit. Phenolic compounds of the different extracts of tucum-do-cerrado peel were identified and quantified using a high-performance liquid chromatography system coupled to a diode array detector (DAD). Total phytochemical compound content was determined by spectrophotometric assays and the antioxidant activity by ferric reducing antioxidant power and β-carotene/linoleic assays. Total phenolic, flavanols, total anthocyanins and yellow flavonoids concentration of tucum-do-cerrado were 122-, 14-, 264- and 61-fold higher in the peel than in the pulp, respectively. The aqueous, methanolic and ethanolic extracts of the tucum-do-cerrado peel exhibited higher antioxidant activity compared to its pulp. Flavanols, anthocyanins, flavones, phenolic acids and stilbenes were the main phenolic classes identified in the tucum-do-cerrado peel extracts. Results suggest that the antioxidant capacity and the phytochemical compound content of the tucum-do-cerrado are mainly associated with the peel. Although flavonoids are the main compounds identified in tucum-do-cerrado peel, other phenolics identified in minor amounts, such as phenolic acids and stilbenes, may be responsible for the high antioxidant capacity of the fruit.

## 1. Introduction

*Bactris setosa* Mart, more commonly called tucum-do-cerrado, is a palm shrub that grows near marshes and semi-shade, reaching up to 4.5 m high and producing an ovoid black-purple fruit 1.1 to 2.1 cm long and 1.3 to 2.2 cm wide [1]. Tucum-do-cerrado fruit has a fibrous peel and succulent pulp, which are consumed fresh by the indigenous population and Cerrado residents, and the whole seed is used for crafts [2]. In a comparative study carried out by our group, tucum-do-cerrado fruit has been identified as one of the four Cerrado fruits having the highest antioxidant activity and phenolic compound content relative to the red delicious apple (*Malus domestica* Borkh) [3]. These results suggest that phenolic compounds could be responsible for the fruit’s antioxidant activity.

The role of bioactive compounds in health maintenance has been extensively reported. Once absorbed by the organism, they can modulate several pathophysiological processes such as inflammation, oxidative stress, blood pressure, microbial growth and mutagenic processes, reducing the risk of diseases [4,5,6]. Among the compounds with antioxidant activity, more than 8000 phenolic compounds were identified in fruits and were classified according to their chemical structure, such as flavonoids (anthocyanins, flavones, flavanols), phenolic acids, stilbenes and tannins [7]. The antioxidant activity of stilbene compounds seems to be associated with the upregulation of the nuclear factor-erythroid-2-related factor-2 (Nrf2), and the consequent upregulation of the Nrf2-regulated cytoprotective genes such as catalase, heme oxygenase-1 (HO-1) and NAD(P)H dehydrogenase, quinone 1 (NQO1) [8]. Anthocyanins, the flavonoids responsible for the blue-purple color of many fruits, seem to protect cells against oxidative stress by inducing glutathione (GSH) synthesis [9]. Some polyphenols have an iron chelator property, and hence, reduce iron availability and improves the antioxidant/oxidant status *in vivo* [10], while others such as the flavonol quercetin, which is one of the most potent natural antioxidants, act as a free radicals scavenger [11]. Although it is known that tucum-do-cerrado contains a high amount of phenolic compounds, the specific classes of phenolics present in this fruit are still unknown. Therefore, the present study aimed to identify the main phytochemical compounds in the tucum-do-cerrado fruit through high performance liquid chromatography coupled with a photodiode detector (HPLC-DAD) and evaluate the part of the fruit with the highest phenolic content and antioxidant activity.

## 2. Experimental Section

### 2.1. Reagents and Standards

Reagents were purchased from Merck (Darmstadt, Germany), Sigma-Aldrich Inc. (Saint Louis, MO, USA) and Fluka^®^ (Steinheim, Germany). Phenolic standards were all purchased from Sigma-Aldrich Inc. (Saint Louis, MO, USA).

### 2.2. Sample Material

Five kilograms (5 kg) of the edible portion of ripened tucum-do-cerrado (*Bactris setosa* Mart) fruits were harvested at a farm localized at Terezópolis de Goiás, 16°28′15.4′′S and 49°03′44.1′′W, Goiás, Brazil. After selection of the fruits, by full maturity and peel integrity, the sample was washed using deionized water and stored at −80 °C until separation of the edible parts. During the separation of the edible parts, one fruit at a time was removed from the liquid nitrogen, and the peel and pulp were manually separated from the seeds, using stainless materials. The entire procedure was conducted in the dark. After separation, the peel or the pulp were powdered in liquid nitrogen, using a porcelain mortar. Aliquots of 400 g of the powdered samples, peel or pulp, were stored at −80 °C until analysis. The fruit was botanically identified at the Botanical Department herbarium in the Biological Institute at the Universidade de Brasília, Distrito Federal, Brazil.

### 2.3. Moisture Content

Five grams of tucum-do-cerrado fruit frozen pulp or peel were ground using liquid nitrogen in a mortar with a porcelain pestle and freeze-dried in a Liotop L101 lyophilizer, São Paulo, Brazil. Moisture content was defined as the difference between the dry weight and the wet weight expressed as the percentage of wet fruit.

### 2.4. Extraction and Isolation

Liquid extractions of peel or pulp were performed using deionized water, 95% ethanol and 1.5 mol/L HCl (85:15) solution and acidified methanol (1% HCl). Peel or pulp (200 g) were ground and the compounds were successively extracted using deionized water. Samples were shaken (1 g) for 1 h at 30 °C and then filtered in JP41 filter paper (JProlab^®^—Alemanha) under vacuum. Peel and pulp aqueous extracts (AqE) were lyophilized, yielding 30.79 and 19.20 g of powder, respectively, and stored at −80 °C until analysis. Three grams of the lyophilized aqueous extract (AqE) of tucum-do-cerrado pulp or peel were diluted in 50 mL of methanol/water solution (1:3, *v/v*), and then subjected to subsequent liquid-liquid partition using hexane and ethyl acetate. Partition included addition of hexane (2 × 50 mL) to the methanol/water (1:3, *v/v*) solution, then the mixture was stirred for 20 min under darkness and allowed to rest until it was in two phases, and the organic layer was collected (hexane fraction—HexF). Subsequently, the methanol/water (1:3) fraction was submitted again to the ethyl acetate liquid-liquid partition procedure as described above, and another organic layer was obtained (ethyl acetate fraction—EAF). The remaining extract was the methanol/water fraction (MAqF). All fractions were stored −80 °C until analysis [12].

Fresh samples of tucum-do-cerrado peel or pulp were also ground and ethanol and methanol extracted. Approximately 2 to 4 g of fresh pulp or peel were ground extracted using 95% ethanol and 1.5 mol/L HCl (85:15) solution (EE) or acidified methanol (1% HCl) (ME). Solutions were allowed to stand at 4 °C for 16 h. Finally, the samples were shaken (1 g) for 1 h at 30 °C and then filtered on JP41 filter paper under vacuum to remove remaining particles. The filter residue was submitted twice to the extraction procedure described above. The volume was made up to 25 mL using its respective solvent. EE and ME were stored at −80 °C until the analysis.

### 2.5. HPLC-DAD Analysis of Phenolic Compounds

Lyophilized AqE and MAqF, HexF and EAF of tucum-do-cerrado peel were dissolved in water (2% formic acid):methanol (0.5% formic acid); 90:10, *v/v*. Peel EE and ME were dissolved in the same solvents. All samples were filtered through 0.45 μm membranes (Millipore^®^, Bedford, MA, USA). All analysis was conducted protected from light and in triplicate.

Phenolic compounds profiles were determined according to the procedure proposed by Simirgiotis, *et al.* [13], with slight modifications. Chromatographic analysis of tucum-do-cerrado extracts were carried out on a Shimadzu^®^ LC-20AD (Kyoto, Japan) HPLC system equipped with a LC-20AD HPLC pump, a SIL-20AD autosampler, a CTO-20AD thermostatted column compartment and a SPD-20AD photodiode array detector. The analytical column was an ODS-CLS C18 column (250 mm × 4.6 mm i.d.; particle size 5 μm, Restek^®^, Bellefonte, PA, USA) protected with a Guard Cartridge (Restek^®^, Bellefonte, PA, USA). The column was maintained at 25 °C. The mobile phase consisted of 2% formic acid solution (A) and methanol: acidified water (0.5% formic acid; 9:1, *v/v*) (B). The elution profile was 0–35 min, 90%–75% A; 35–65 min, 75%–40% A; 65–70 min, 40%–90% A; 70–75 min, 90% A. A 1 mL/min flow rate was used and 20 to 50 μL of samples were injected. Spectral data were recorded from 200 to 700 nm during the whole run.

Phenolic compounds were identified by comparing their retention time and UV–visible spectral data to previously injected standards. Compounds quantification was done by external standardization. Calibration was performed by injecting the standard working solution in triplicate at five different concentrations for each compound, based on its expected content ranges in the samples. All standard curves were linear in the concentration ranges expected in the samples and had coefficients of determination ranging from 0.9932 (for anthocyanin) to 0.9999 (caffeic acid and resveratrol).

### 2.6. Antioxidant Activity

#### 2.6.1. Ferric Reducing Antioxidant Power Assay (FRAP)

The antioxidant activity of different extracts was estimated by FRAP assay [14], with modifications as described previously by Siqueira *et al.* [3].

#### 2.6.2. Carotene/Linoleic System

The antioxidant activity of each extract was estimated spectrophotometrically based on the β-carotene discoloring induced by the oxidative degradation of linoleic acid [15].

### 2.7. Determination of Phytochemical Compounds

The total polyphenols (TP) in the tucum-do-cerrado whole fruit, pulp and peel extracts was determined according to the Folin-Ciocalteu method [16].

The total flavanol (TFA) concentration in tucum-do-cerrado extracts was determined using the chromogen p-dimethylaminocinnamaldehyde (DMACA) method [17].

Total anthocyanins and yellow flavonoids were determined according to a spectrophotometric method proposed by Francis [18]. Results were expressed in mg of compound per 100 grams of fresh fruit.

Tucum-do-cerrado pulp and peel ascorbic-acid (AA) content was determined using the 2,6-dichlorophenol-indophenol (DCIP) titration method, according to AOAC procedure [19].

Total carotenoids were extracted and quantified using the method described by Rodrigues-Amaya [20].

### 2.8. Statistical Analysis

A *t* test for independent samples was used to make comparisons between the mean values of the extracts of whole fruit, peel and pulp and between the mean values of each fraction of the extracts. The correlation between the values of the phytochemical compounds and the antioxidant activity were analyzed using the Pearson test. The analysis was performed using the SPSS Statistics 17.0 program (SPSS Inc., Chicago, IL, USA). Significance was defined as *p* < 0.05 and the variables are presented as the mean ± SD.

## 3. Results and Discussion

In a previous study by our group, the tucum-do-cerrado fruit was identified as a Brazilian Savanna fruit with higher antioxidant activity and bioactive compounds content than the Red Delicious apple [3]. In order to evaluate phytochemical compounds composition in tucum-do-cerrado, the present study aimed to identify and quantify the major phenolic compounds present in the pulp and the peel of the fruit, as well as to evaluate antioxidant activity in each portion.

### 3.1. Tucum-Do-Cerrado Peel Has Higher Total Phenolic, Total Flavanol and Total Anthocyanin Content than the Pulp

Tucum-do-cerrado pulp or peel extract moisture content; total phenolics, flavanols and anthocyanins; yellow flavonoids; vitamin C; and carotenoid concentrations are presented in Table 1. Due to the peel’s fibrous texture, its moisture content was lower than that obtained for the tucum-do-cerrado pulp (71.43% ± 0.84% and 91.23% ± 1.26%, respectively). A similar moisture content was obtained for the fresh jaboticaba peel (79.5%) [21], a fruit that resembles tucum-do-cerrado. Phenolic compound concentration and vitamin contents were higher in peel when compared to pulp, suggesting that peel is the main contributor of phytochemical compounds content of tucum-do-cerrado fruit (Table 1). The result is consistent with data previously reported indicating that peel tissues usually contain more phenolic compounds than flesh tissues [22,23]. Among the classes of phytochemical compounds analyzed, total phenolics, flavanols and anthocyanins represented the major classes in the tucum-do-cerrado peel, while in the pulp only the first two classes of compounds had high concentrations. Notably, the phenolics, flavanols, anthocyanins and yellow flavonoids compound concentrations were 122-, 14-, 264-, and 61-fold higher in the peel than in the pulp. The greater exposure of the peel to environmental stress may explain the highest concentration of phenolic in it, as the outer portion of the fruit, it provides greater protection. Phenolic compounds may act as protective agents against UV lights, pathogens and predators in fruits and vegetables [24].

The total phenolic levels found in tucum-do-cerrado (whole fruit) were higher than those obtained for other six non-traditional Brazilian tropical fruits, açaí (*Euterpe oleracea*), camu-camu (*Myrciaria dubia*), jaboticaba (*Myrciaria cauliflora*), jambolão (*Syzygium cumini*), juçara (*Euterpe edulis*) and murta (*Blepharocalyx salicifolius*) (185 to 1176 mg gallic acid equivalent/100 g of fresh fruit) [25], which resemble tucum-do-cerrado fruit; all of them are purple-colored fruits. Souza *et al.* [26] classified fruits into three categories based on their polyphenol content: low (<100 mg gallic acid equivalent/100 g), medium (100–500 mg gallic acid equivalent/100 g) and high (>500 mg gallic acid equivalent/100 g); the tucum-do-cerrado should be considered a fruit with high phenolic content.

Total flavanol content found in tucum-do-cerrado was about 5 to 107-fold (7 to 91 mg CE/100 g of fresh fruit) higher than the content reported for jambolão [25,27]. The wide range of total flavanols obtained for jambolão may be attributed to the inherent variability of the raw material, as well as to differences between the applied methodologies [27].

Although tucum-do-cerrado fruit showed similar levels of total anthocyanins compared to other purple-colored tropical fruits (açaí, camu-camu, jaboticaba and jambolão; 42–111 mg anthocyanins/100 g fresh fruit), even higher values were obtained for juçara and murta, ranging from 140 to 200 mg/100 g of fresh fruit [25]. In contrast, tucum-do-cerrado peel exhibited an extremely high content of total anthocyanins compared to the above fruits. In relation to yellow flavonoid content, tucum-do-cerrado cannot be considered a source of yellow flavonoid compounds when compared to other similar studied tropical fruits, such as açaí, jambolão, juçara and murta. The mentioned fruits showed a content of 20.1 to 375 mg/100 g fresh fruit, at least 2-fold greater than the one obtained for tucum-do-cerrado fruit in the present study.

Although tucum-do-cerrado fruit showed similar content of vitamin C in relation to other tropical fruits such as açaí, caju, carnaúba, jambolão, juçara, mangaba, murici and murta (78 to 190/100 g fresh fruit) [24], it can not be considered the highest source of vitamin C, as some tropical fruits such as acerola and camu-camu have amounts much higher (1357 and 1882 mg/100 g FW) than that obtained for tucum-do-cerrado.

Carotenoids have been identified not only as compounds with significant antioxidant activity but also as important precursors of vitamin A in plant foods. The total carotenoid content of tucum-do-cerrado was determined and the results expressed as β-carotene equivalent, as showed in Table 1. According to literature data, tucum-do-cerrado should not be considered a potential source of β-carotene, once the concentration found was lower than 20 μg of β-carotene/g [28]. The carotenoid concentration in the tucum-do-cerrado was significantly lower when compared to fruits considered sources of this nutrient, such as buriti palms, tucumã, bocaiuva, bacuri and umari (mari); values ranged from 99 to 364 μg of β-carotene/g of fresh fruit [28]. Mangaba and gurguri, non-traditional Brazilian fruits, also presented total carotenoid content (300 μg to 4700 μg total carotenoids/100 g fresh matter) higher than tucum-do-cerrado fruit [24]. Thus, carotenoids should not be the main phytochemical responsible for the high antioxidant activity of the tucum-do-cerrado.

### 3.2. Tucum-Do-Cerrado Peel has a High Antioxidant Activity via Ferric Reducing Antioxidant Power (FRAP) and β-Carotene/Linoleic Acid Assays in Relation to Pulp

A recent study of our group demonstrated that ethanolic and aqueous extracts of tucum-do-cerrado had one of the highest antioxidant activities among twelve Cerrado fruits evaluated by FRAP assay [3].

To obtain extracts with different chemical composition and therefore identify the fractions which contain the main compounds responsible for the antioxidant potential of the tucum-do-cerrado, the peel and pulp samples were submitted to extraction using solvents that present different polarities. In the present study, the ethanolic (EE) and methanolic (ME) extracts of peel or pulp of tucum-do-cerrado exhibited the highest antioxidant activity according to the FRAP assay when compared to its own aqueous extract (AqE; Table 2). Among aqueous extract fractions, the methanol/water fraction (MAqF) had higher antioxidant activity compared to ethyl acetate (EAF) and hexane (HF) fractions for both peel and pulp extracts (Table 2). Differences in the antioxidant activities obtained among the tucum-do-cerrado extracts may be attributed to differences in their phytochemical compound content as well as in the type of compound, which in turn depend on the used solvent polarity.

Tucum-do-cerrado extract antioxidant activity was also evaluated by the β-carotene/linoleic assay. The results, expressed as the inhibition percentage of β-carotene oxidation, are shown in Table 2. Following the trend observed in the FRAP assay, tucum-do-cerrado peel or pulp ME and EE extracts inhibited the oxidation of β-carotene to a greater extent than AqE, and the peel extracts also had greater antioxidant activity compared to pulp. As showed in Table 2, peel or pulp hexane fraction (HexF) showed almost no antioxidant activity in the FRAP and β-carotene/linoleic acid assays. Together, these results showed that the antioxidant activity of the tucum-do-cerrado fruit should be mainly associated with compounds present in the peel, which, in their majority, are soluble in polar organic solvents than in hexane or organic apolar solvents. It is important to consider that the AqE was lyophilized, while in the ME and EE, the solvent was evaporated under a nitrogen stream; therefore, the lower antioxidant activity of the AqE compared to the other extracts may be associated with losses of volatile antioxidant compounds due to the lower pressure used in the lyophilization process, which may have resulted in the impairment of AqE antioxidant activity.

The antioxidant activities of AqE, ME and EE extracts of the tucum-do-cerrado peel, estimated by FRAP assay (160.36 to 303.90 μmol Fe_2_SO_4_/g fruit), were higher than antioxidant activities of fruits commonly consumed by the world population such as avocado, banana, grape, orange, papaya, pineapple and watermelon, whose values ranged between 2.76 to 14.5 μmol Fe_2_SO_4_/g fruit [29]. Even the values of ME and EE of tucum-do-cerrado pulp, which showed low antioxidant activity compared to the peel extracts, were at least 1.5-fold higher than those conventional fruits. These results demonstrate a high antioxidant activity of tucum-do-cerrado fruit. However, the antioxidant activities of the AqE, ME and EE extracts of tucum-do-cerrado peel and pulp, determined by the β-carotene/linoleic acid assay, were below that obtained for some tropical fruits of Brazil (32% to 91% g fresh fruit).

An early study, involving eighteen non-traditional tropical fruits from Brazil, açaí, camu-camu, jaboticaba, jambolão, juçara and murta, which have physical characteristics similar to the tucum-do-cerrado, showed antioxidant activity significantly lower (32.1–279 μmol Fe_2_SO_4_/g of fresh fruit [25] than that obtained for tucum-do-cerrado peel extracts.

Despite the low vitamin C and carotenoids levels of tucum-do-cerrado fruit compared to the total phenolic content, significant positive correlations were found between vitamin C and total carotenoids and the antioxidant activity of the ethanolic extract measured by FRAP and β-carotene/linoleic acid assays (*r* = 0.9784 *p* = 0.001, *r* = 0.9964 *p* < 0.0001, for FRAP assay and *r* = 0.9900 *p* < 0.0001; *r* = 0.9888 *p* < 0.0001, for β-carotene/linoleic acid assays, respectively), as well as for total phenolic content and these antioxidant assays (*r* = 0.9997 *p* < 0.0001 for FRAP and *r* = 0.9972 *p* < 0.0001 for β-carotene/linoleic acid assays). The high concentration of antioxidant compounds found in the tucum-do-cerrado peel compared to its pulp content might be related to the direct contact of peel with environmental damage. Probably adverse abiotic factors, such as soil acidity, excessive exposure to sunlight and frequent fire during the dry season may have stimulated the plant to synthesize antioxidant compounds to protect it against oxidative stress, and consequently, those resistant species were selected in this Biome Cerrado. Furthermore, several abiotic stresses such as wounding, UV-light, hyperoxia, exogenous application of ethylene, or nutrient deficiency in the growing medium may significantly increase the concentration of polyphenols and other bioactive compounds in fruits and vegetables, as well as their antioxidant capacity [30,31].

### 3.3. Flavanols, Anthocyanins, Hydroxybenzoic Acids and Flavones Are the Major Phenolics Compound Classes Identified in Tucum-Do-Cerrado Peel by HPLC-DAD

**Figure 1:**
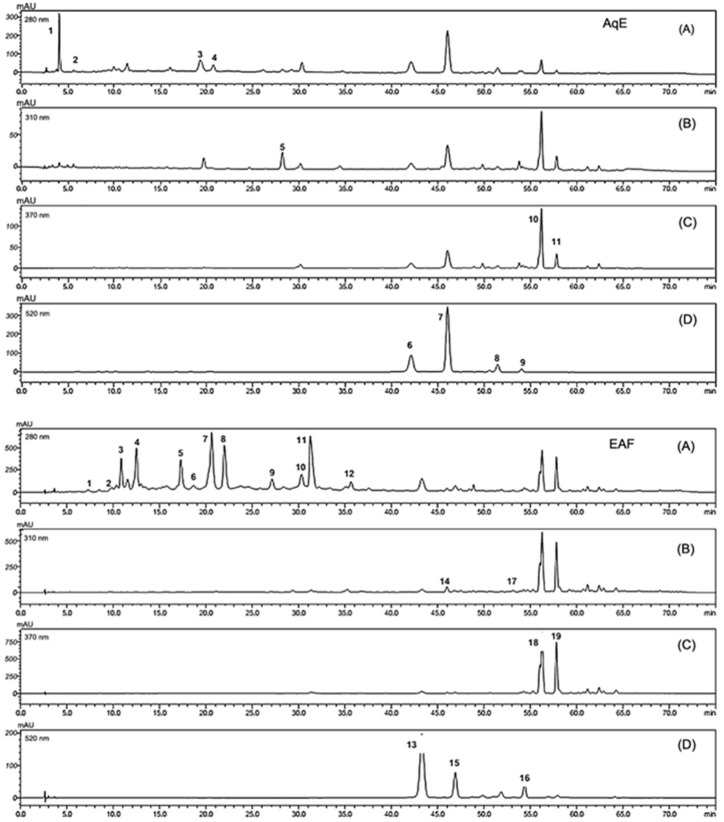

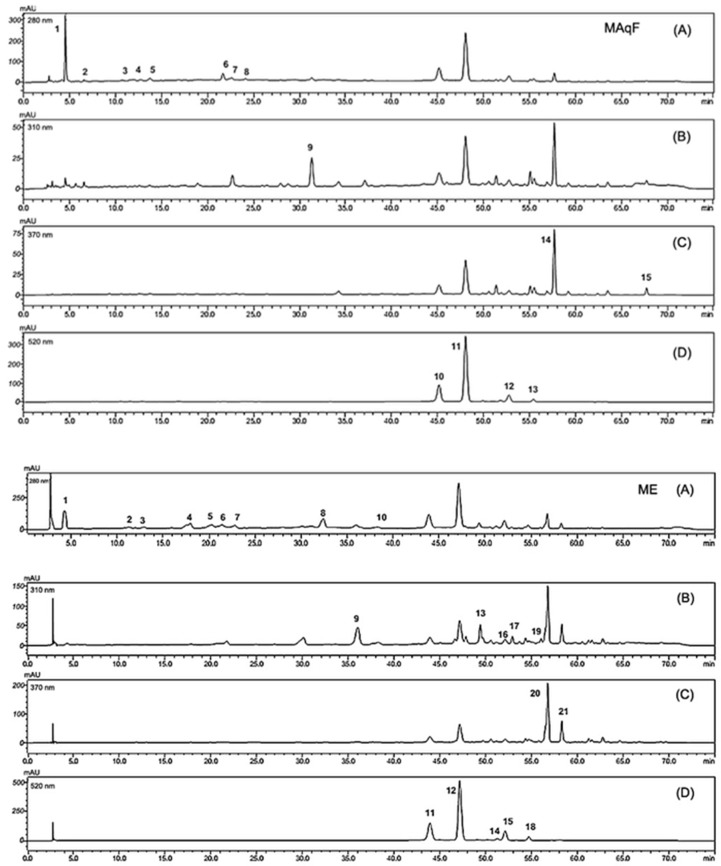

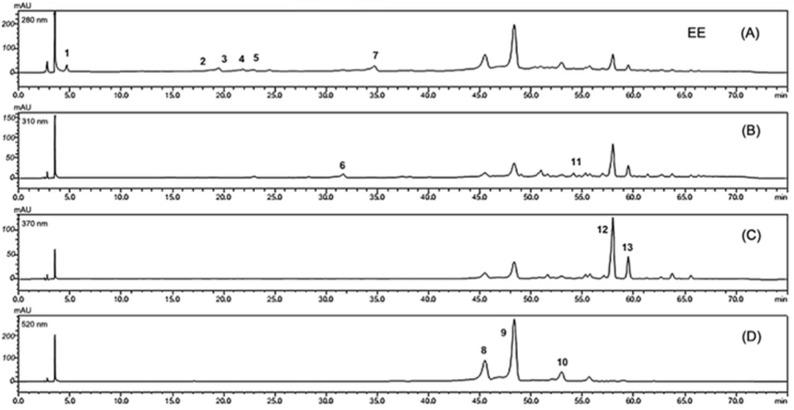
Chromatograph profile of tucum-do-cerrado peel extracts. (AqE) lyophilized aqueous extract; (EAF) lyophilized ethyl acetate fraction solubilized in water and methanol (9:1); (MAqF) lyophilized methanol/water fraction solubilized in water and methanol (9:1); (ME) methanol extract (1% HCl); (EE) ethanol extract (1.5 mol/L HCl). Chromatographic conditions: column C18; mobile phase deionized water:formic acid 9:1 (*v/v*); injection volume 20 μL; and spectrophotometer detection at 280 nm (**A**); 310 nm (**B**); 370 nm (**C**) 520 nm (**D**).

The analytical HPLC-DAD method was optimized with the aim to obtain an efficient chromatographic separation, identification and quantification of the main components of the different extracts of tucum-do-cerrado peel. The HPLC chromatograms recorded at 280 nm (hydroxybenzoic acids—gallic acid and flavanols—catechin), 310 nm (hydroxycinnamic acids—caffeic and ferulic acids and stilbenes—trans resveratrol), 370 nm (flavones—rutin and flavonols—quercetin) and 520 nm (anthocyanins—cyanidin) nm for aqueous extract (AqE) and its fractions, EAF and MAqF, and for the methanolic and ethanolic extracts, are shown in Figure 1.

In most of the analyzed tucum-do-cerrado peel extracts, three major classes of phenolic compounds were identified: flavonoids, phenolic acids and stilbenes. Among the flavonoids, flavanols, anthocyanins, flavones and flavonols were identified. The phenolic acids identified were hydroxybenzoics and hydroxycinnamics acids. The stilbene class was identified in minor content and only in tucum-do-cerrado peel alcoholic extracts. The phenolic compound identification was done based on the retention time and the UV-Vis profile of the external standard compounds.

### 3.4. Main Classes of Phenolic Compounds Identified and Quantified in Aqueous, Methanolic and Ethanolic Extracts of Tucum-Do-Cerrado Peel by HPLC-DAD

The most abundant classes of phenolic compounds identified in the tucum-do-cerrado peel aqueous extract were flavanols and anthocyanins, followed by flavones and hydroxybenzoic acids (Table 3). The chromatogram profile of the ethyl acetate fraction (EAF) showed the best separation among the other fractions, composed mainly by the flavonoids. Partition offered efficient flavonoid isolation. The flavanols represented 91% of phenolic compounds identified, followed by the flavones (Table 3). Anthocyanins, phenolic acids and stilbenes were detected in very low concentrations in EAF. Krasteva *et al.* [32] reported that flavones (rutin) and flavonols (quercetin and kaempferol) have high affinity for ethyl acetate due to their hydrophobic character.

Jaboticaba (*M. jaboticaba* (Vell.) *Berg*.), a Brazilian purple-peel fruit that exhibits similar characteristics to tucum-do-cerrado, also presented substantial concentration of anthocyanins, delphinidin 3-glucoside and cyanidin 3-glucoside (634.75 ± 1.82 and 1963.57 ± 52.72 mg/100 g of freeze-dried jaboticaba powder) in the peel [21].

In a recent study, carried out on an Amazonian palm *Oenocarpus bataua* (“patawa”) which produces a fruit with purple peel similar to the tucum-do-cerrado, anthocyanins, condensed tannins, stilbenes and phenolic acids were identified as the main phenolic compounds classes present in its pulp. The authors found a stronger antioxidant activity via FRAP assay [33], similar to that obtained in the present study for tucum-do-cerrado. Similar phenolic composition to that of tucum-do-cerrado peel was also found in the jussara palm plant (*Euterpe edulis*), a purple-peel fruit from the Atlantic Forest located in southern of Brazil. The phenolic compounds identified in jussara fruit included ferulic, gallic, and p-coumaric acids, as well as catechin, epicatechin, quercetin and anthocyanins [34].

A high content of flavanols (75.89 mg catechin derivative/100 g fresh weight; 48% of the identified compounds) was detected in both the methanol:water (MAqF) fraction and EAF fraction (Table 3). However, contrary to that observed for the EAF fraction, the MAqF also presented a substantial concentration of anthocyanins and hydroxybenzoic acids (40.93 cyanidin derivative and 28.42 gallic acid derivative mg/100 g fresh weight, respectively; 26% and 18% of the identified class of compound). Such compounds can be in the form of gallic acid salts and esters. These compounds have high solubility in alcoholic solutions such as methanol and ethanol [7].

Among the evaluated tucum-do-cerrado peel extracts, the methanol extract presented the highest concentration of identified phenolic compounds classes (1104.01 mg/100 g fresh weight) compared to aqueous and ethanol extracts (541.22 and 543.44 mg/100 g fresh weight, respectively). Results suggest that the freeze-drying process used to obtain the AqE reduced the phenolic content of tucum-do-cerrado peel. In addition, the chromatographic profile changed according to the drying process; for example, the lyophilization process used to obtain the AqE led to a higher loss of flavanols, flavones and anthocyanins (2.4-, 1.8- and 1.6-fold, respectively) relative to the ME. However, the hydroxybenzoics acid content did not change between the two extracts. Garcia-Salas *et al.* [35] observed that freeze-dried lemon powder had lower phenolic content than the low vacuum-dried-lemon.

Mulinacci *et al.* [36] observed that the phenolic compounds extraction from rosemary leaves using ethanol/water yielded lower amounts of rosmarinic acid and a rapid degradation of carnosic acid toward its oxidized form when compared with the ethanol extract. The authors also suggest that some phenolic compounds are very susceptible to oxidation reactions and consequently very unstable in an aqueous environment due to the high activity of phenoloxidase. The authors also observed a reduction in the flavonoid content of freeze-dried rosemary leaves extract in relation to fresh leaves extract, mainly the glycosylated forms. However, the freeze-drying process retained the highest amount of total phenolics, anthocyanins, total flavonoids and antioxidant activity compared to other drying methods, such as ambient air drying, vacuum at 40 °C and oven at 40 °C, as demonstrated by Tseng & Zhao [37] in wine grape pomace samples (Pinot Noir and Merlot).

Thus, considering that in the present study, the ethanol extract was prepared in a similar manner as methanol extract, the lower phenolic content obtained in ethanol extract may be explained by the different solubility of the compounds to each solvent. The solubility of the polyphenol depends mainly on its number of hydroxyl groups, the molecular size and the length of the hydrocarbon chain [38].

Despite the highest phenolic composition obtained on the methanolic extract, it showed lower antioxidant activity compared to ethanolic extract of tucum-do-cerrado peel. The results suggest that the antioxidant activity of the extract should be related to the type of phytochemical rather than its concentration. Stilbenes (expressed as trans resveratrol derivative) were identified only in tucum-do-cerrado peel methanolic and ethanolic extracts, and were found in lower concentrations compared to the other phenolics. The higher antioxidant activity in these two extracts than aqueous extracts may be related to stilbene presence (Table 3).

## 4. Conclusions

In conclusion, phenolic compounds are the main phytochemical molecules of tucum-do-cerrado peels. Regarding the different phenolic classes identified, flavanols, anthocyanins and flavones are the major classes, followed by hydroxybenzoic acids, hydroxycinnamic acids and stilbenes, in minor concentrations. The higher antioxidant activity of tucum-do-cerrado peels must be attributed to their higher content of phytochemical compounds in relation to the pulp, in particular its polyphenols composition. Further studies with tucum-do-cerrado are being developed in our laboratory, with the aim of analyzing the antioxidant activity *in vivo* of this little-known fruit produced in Cerrado Biome. We suppose that tucum-do-cerrado fruit may provide a source of new phytochemical compounds with functional properties beneficial to health, which should stimulate the pharmaceutical and food industries for the development of new products. New knowledge about the potential of the Cerrado flora will, certainly, promote the sustainable development of regions that have similar climatic edaphic conditions to the savannas of Africa.

## Figures and Tables

**Table 1:** Moisture content (%) and phytochemical compounds (mg/100 g) of tucum-do-cerrado peel, pulp and whole fruit expressed as fresh matter.

Components	Tucum-Do-Cerrado		
	Peel	Pulp	Whole Fruit
Moisture	71.4 ± 0.8 **^;##^	91.2 ± 1.3 ***	77.9 ± 1.0
Total phenolics ^§^	28,287.6 ± 614.8 ***^;##^	230.5 ± 0.9 ***	5214.3 ± 132.5
Total flavanols ^§^	1068.5 ± 10.2 **^;##^	79.2 ± 2.0 ***	746.7 ± 4.6
Total anthocyanins	638.5 ± 3.3 ***^;##^	2.4 ± 0.1 **	83.2 ± 6.5
Yellow flavonoids	323.0 ± 7.9 **^;##^	5.3 ± 0.1 ***	42.2 ± 2.1
Vitamin C	181.2 ± 23.2 *^;#^	53.7 ± 0.0 *	100.6 ± 20.3
Total carotenoids ^¥^	290.4 ± 20.7 **^;##^	56.5 ± 4.7 **	147.8 ± 20.5

Means ± SD (*n* = 3) following by *; **; *** are significantly different from values obtained for whole fruit and followed by ^#; ##; ###^ are significantly different from pulp (*^;#^ *p* < 0.05; ** *p* < 0.01; ***^;##^ *p* < 0.001); *t*-test, independent samples. ^§^ Total phenolic and total flavanols results were expressed as mg of gallic acid equivalent and mg of catequin equivalent/100 g, respectively; ^¥^ Total carotenoids were expressed as mg of β-carotene (μg/100 g).

**Table 2:** Antioxidant activity of peel or pulp of tucum-do-cerrado extracts.

Extracts	FRAP (μmol Fe_2_SO_4_/g Fresh Matter)	β-Carotene/Linoleic Acid (%) g Fresh Matter
Peel		
Aqueous extract (AqE)	160.36 ± 9.48	3.13 ± 0.10
Methanol/water fraction (MAqF)	68.02 ± 0.74 *	1.12 ± 0.11 ***
Ethyl acetate fraction (EAF)	37.09 ± 0.55 *^;§§§^	0.61 ± 0.04 ***^;§§^
Hexane fraction (HF)	2.69 ± 0.09 *^;§§§^	nd
Methanolic extract (ME)	237.95 ± 6.50 **	3.41 ± 0.10 *
Ethanolic extract (EE)	303.90 ± 12.52 **	7.04 ± 0.42 ***
Pulp		
Aqueous extract (AqE)	9.66 ± 0.06 ^##^	0.33 ± 0.08 ^###^
Methanol/water fraction (MAqF)	3.01 ± 0.06 **^;^^###^	0.15 ± 0.06 *^;^^###^
Ethyl acetate fraction (EAF)	2.39 ± 0.07 **^;^^###^^;§§§^	0.08 ± 0.03 **^;^^###^
Hexane fraction (HF)	0.22 ± 0.01 **^;^^###^^;§§§^	nd
Methanolic extract (ME)	26.92 ± 0.43 **^;^^###^	0.67 ± 0.04 **^;^^###^
Ethanolic extract (EE)	22.81 ± 1.16 *^;^^###^	0.91 ± 0.14 **^;^^###^

Means ± SD (*n* = 3) following by *; ** are significantly different from values obtained for aqueous extract of peel or pulp, and followed by ^#; ##; ###^ are significantly different from values obtained for peel at the same extract or subfraction (*^;#^ *p* < 0.01; **^;##^ *p* < 0.001;); *t*-test, independent samples. nd, non determined; ^§^ values are significantly different from that obtained for methanol/water fraction.

**Table 3:** Main classes of phenolic compounds identified and quantified in different extracts of tucum-do-cerrado peel by HPLC-DAD.

	Peak Number	Retention Time (min)	λ Max. (nm)	Compound Class	Hypothesis	Amount (mg/100 g FW)
Aqueous extract (AqE)						
	1	4.05	226, 272	Hydroxybenzoic acid	Gallic acid derivative	61.46
	2	5.60	224, 276	Hydroxybenzoic acid	Gallic acid	0.65
	3	19.28	224, 278	Flavanol	Catechin derivative ^a^	214.61
	4	20.70	224, 278	Flavanol	Catechin derivative ^a^	88.97
	5	28.16	222, 319	Hydroxycinnamic acid	Caffeic acid	1.95
	6	42.05	279, 516	Anthocyanin	Cyanidin derivative ^b^	25.13
	7	45.99	280, 520	Anthocyanin	Cyanidin derivative ^b^	71.44
	8	51.41	279, 520	Anthocyanin	Cyanidin derivative ^b^	6.52
	9	54.03	279, 520	Anthocyanin	Cyanidin derivative ^b^	1.74
	10	56.13	234, 355	Flavone	Rutin	60.56
	11	57.77	257, 355	Flavone	Rutin derivative ^d^	8.19
EAF						
	1	7.25	226, 278	Hydroxybenzoic acid	Gallic acid derivative	0.93
	2	10.81	224, 278	Flavanol	Catechin derivative ^a^	17.22
	3	11.50	225, 278	Flavanol	Catechin derivative ^a^	6.65
	4	12.46	226, 278	Flavanol	Catechin derivative ^a^	29.09
	5	17.23	225, 278	Flavanol	Catechin derivative ^a^	26.31
	6	18.62	225, 278	Flavanol	Catechin derivative ^a^	3.93
	7	20.56	227, 278	Flavanol	Catechin derivative ^a^	62.85
	8	21.94	226, 278	Flavanol	Catechin derivative ^a^	38.72
	9	27.08	225, 278	Flavanol	Catechin derivative ^a^	9.44
	10	30.27	225, 278	Flavanol	Catechin derivative ^a^	14.49
	11	31.21	226, 278	Flavanol	Catechin derivative ^a^	50.26
	12	35.61	225, 278	Flavanol	Catechin derivative ^a^	4.95
	13	43.26	279, 516	Anthocyanin	Cyanidin derivative ^b^	1.56
	14	45.97	258, 324	Hydroxycinnamic acid	Ferulic acid derivative	0.25
	15	46.86	279, 519	Anthocyanin	Cyanidin derivative ^b^	0.43
	16	51.82	277, 521	Anthocyanin	Cyanidin derivative ^b^	0.06
	17	53.08	300, 314	Stilbene	Resveratrol derivative ^c^	0.04
	18	56.22	255, 354	Flavone	Rutin	13.80
	19	57.77	255, 354	Flavone	Rutin derivative ^d^	8.11
MAqF						
	1	4.50	225, 272	Hydroxybenzoic acid	Gallic acid derivative	27.79
	2	6.54	224, 276	Hydroxybenzoic acid	Gallic acid derivative	0.63
	3	11.87	224, 279	Flavanol	Catechin derivative ^a^	11.07
	4	12.71	224, 279	Flavanol	Catechin derivative ^a^	5.15
	5	13.72	224, 278	Flavanol	Catechin derivative ^a^	12.17
	6	21.66	224, 272	Flavanol	Catechin derivative ^a^	28.32
	7	22.60	224, 280	Flavanol	Catechin derivative ^a^	12.30
	8	24.11	224, 278	Flavanol	Catechin derivative ^a^	6.88
	9	31.31	305, 317	Stilbene	Resveratrol derivative ^c^	0.73
	10	45.15	279, 517	Anthocyanin	Cyanidin derivative ^b^	8.52
	11	48.03	280, 520	Anthocyanin	Cyanidin derivative ^b^	29.34
	12	52.76	279, 521	Anthocyanin	Cyanidin derivative ^b^	2.62
	13	55.47	268, 519	Anthocyanin	Cyanidin derivative ^b^	0.45
	14	57.69	255, 355	Flavone	Rutin derivative ^d^	10.98
	15	67.73	258, 367	Flavonol	Quercetin	1.26
Methanolic extract (ME)						
	1	4.25	224, 272	Hydroxybenzoic acid	Gallic acid derivative	74.30
	2	11.23	224, 278	Flavanol	Catechin derivative ^a^	36.16
	3	12.94	224, 278	Flavanol	Catechin derivative ^a^	27.77
	4	17.96	224, 278	Flavanol	Catechin derivative ^a^	182.32
	5	20.30	224, 278	Flavanol	Catechin derivative ^a^	82.59
	6	21.39	224, 278	Flavanol	Catechin derivative ^a^	57.15
	7	22.79	224, 278	Flavanol	Catechin derivative ^a^	57.44
	8	32.41	224, 278	Flavanol	Catechin derivative ^a^	243.89
	9	35.99	224, 320	Hydroxycinnamic acid	Caffeic acid derivative	7.15
	10	38.29	224, 279	Flavanol	Catechin derivative ^a^	41.63
	11	43.88	279, 516	Anthocyanin	Cyanidin derivative ^b^	37.50
	12	47.13	280, 519	Anthocyanin	Cyanidin derivative ^b^	105.38
	13	49.04	224, 321	Hydroxycinnamic acid	Ferulic acid derivative	4.11
	14	51.21	279, 519	Anthocyanin	Cyanidin derivative ^b^	1.59
	15	52,10	279, 519	Anthocyanin	Cyanidin derivative ^b^	14.85
	16	52.89	307	Stilbene	Resveratrol derivative ^c^	0.83
	17	53.67	225, 323	Hydroxycinnamic acid	Ferulic acid derivative	0.58
	18	54.68	280, 519	Anthocyanin	Cyanidin derivative	5.98
	19	56.02	253, 330	Hydroxycinnamic acid	Ferulic acid derivative	0.94
	20	56.76	255, 354	Flavone	Rutin	95.82
	21	58.29	256, 354	Flavone	Rutin derivative ^d^	26.03
Ethanolic Extract (EE)						
	1	4.68	224, 273	Hydroxybenzoic acid	Gallic acid derivative	8.37
	2	19.52	224, 278	Flavanol	Catechin derivative ^a^	106.84
	2	21.88	224, 278	Flavanol	Catechin derivative ^a^	45.95
	4	22.90	224, 279	Flavanol	Catechin derivative ^a^	18.96
	5	24.48	224, 278	Flavanol	Catechin derivative ^a^	13.67
	6	31.66	305	Stilbene	Resveratrol derivative ^c^	1.72
	7	34.70	224, 278	Flavanol	Catechin derivative ^a^	126.40
	8	45.50	279, 517	Anthocyanin	Cyanidin derivative ^b^	32.73
	9	48.35	280, 520	Anthocyanin	Cyanidin derivative ^b^	83.25
	10	52.97	279, 519	Anthocyanin	Cyanidin derivative ^b^	9.22
	11	54.14	303	Stilbene	Resveratrol derivative ^c^	0.62
	12	57.98	255, 355	Flavone	Rutin derivative ^d^	78.74
	13	59.47	255, 355	Flavone	Rutin derivative ^d^	16.97

AqE, aqueous extract; EAF, ethyl acetate fraction in a solution of methanol:water (1:9); MAqF, methanol:water (1:3) fraction dissolved in a solution of methanol:water (1:9); ME, methanolic extract; EE, ethanolic extract. ^a^ Quantified as catechin; ^b^ Quantified as cyanidin chloride; ^c^ Quantified as trans resveratrol; ^d^ Quantified as rutin trihydrate.
